# Supplementary material for: Development of a computer-aided design software for the quantitative evaluation of aesthetic damage
Source: PLoS One. 2019 Dec 18;14(12):e0226322. doi: 10.1371/journal.pone.0226322 (PMC6919621; doi:10.1371/journal.pone.0226322)
Supplement: S1 Code site — 1. (ZIP) [file pone.0226322.s001.zip › [EESC_jr][Projeto]Relatorio_07_21/reports/templates_zip/medialoot-lumino-admin-bootstrap-template.zip!/Lumino Admin Bootstrap Template/lumino/forms.html]

Lumino - Forms


Toggle navigation

LuminoAdmin

- User 
  - Profile
  - Settings
  - Logout

- Dashboard
- Widgets
- Charts
- Tables
- Forms
- Alerts & Panels
- Icons
- Dropdown
  - Sub Item 1
  - Sub Item 2
  - Sub Item 3
- Login Page

2. Icons

# Forms

Form Elements

Text Input

Password

Remember me

File input


Example block-level help text here.

Text area

Validation

Checkboxes

Checkbox 1

Checkbox 2

Checkbox 3

Checkbox 4

Radio Buttons

Radio Button 1

Radio Button 2

Radio Button 3

Radio Button 4

Selects

Option 1
Option 2
Option 3
Option 4

Multiple Selects

Option 1
Option 2
Option 3
Option 4

Submit Button
Reset Button
